# A Surgical Emergency

**Published:** 1898-09

**Authors:** H. H. Grant


### Selections and Abstracts.

---

### A SURGICAL EMERGENCY.

Even if we are not content with such statistics as prove the curability of carcinoma in any constant ratio, there is a certainty that its manifestations are so manageable in the developing stages as to fairly constitute its early recognition and treatment a surgical emergency. Beyond any question every manifestation of malignant tendency had at one time a local origin. Such a statement, indeed, is true of all causes. It is peculiarly true of carcinoma that in certain situations this localization remains much longer than in others, and as a result the lesion is much more susceptible of cure in one part of the body than in another. It is only fair to remark in passing that individual resistance in this lesion, as in many others, is a noticeable feature in its progress.

Given two cases of carcinoma of six months' duration, one may live three years, while the other may perish in twelve or fifteen months. One may after operation remain well five to ten years, while the other will present a rapid and fatal recurrence. Notwithstanding this is true of carcinomas in all situations of the body, it is nevertheless a fact that in certain regions the local manifestation is much more easily and thoroughly removed than in others, and beyond all question prompt operative steps in the early stages add many years of life and comfort. So well established are the facts to which we refer that the simple statement is enough, without reference to statistics with which all interested as operators are familiar. Authorities differ and statistics vary, but upon this point concurrence is practically a unit. It is the object of these notes to direct the attention of the family physician anew to the conclusions of the surgeon in respect to the prompt recognition and treatment of certain forms of carcinoma. No confusion can arise in the discussion of this subject if it is definitely understood that when carcinoma has become constitutional—*i. e.*, where secondary deposits which are not susceptible of removal, or where a general cachexia exists—a cure can not be hoped for, though even here in some instances palliative operative steps may often be instituted. Nor must the fact be lost sight of that just when the line of hopelessness is passed is by no means easy to determine in all cases. If, then, in a review of these facts we appreciate that local carcinoma, if thoroughly removed, has no more tendency to return than to originally develop; that the hour when such a growth can be removed with certainty is as near as possible to its discovery; that the mortality of such operation is practically nil; and that death is *certain* in a painful and often loathsome form without it, the responsibility of an early diagnosis and prompt aggressive treatment admits of no discussion. The field is far too large for extended consideration, and besides, the practical fruits of study are limited usually to the accuracy and simplicity of the facts determined. Hence it is profitable, perhaps, to avoid all disputed points; but to take positive data when obtainable. Three forms of carcinoma serve so well to illustrate the importance of this lesion that others will not be now looked into —*epithelioma* of the *lip*, of the *penis*, and *scirrhus of the mammary gland*. These are perhaps the most common forms of external carcinoma; they are so readily discovered and diagnosticated as to leave no excuse for delay; that they are curable if attacked early in from twenty to forty per cent. has been demonstrated so certainly as to entitle them to operation as clearly as an acute abscess; delay offers no prospect of relief. The conclusion is then unmistakable.

The mammary gland, besides presenting in scirrhus the classical symptoms of a tumor—painful and gradually becoming fixed; usually after the thirty-fifth year; most commonly in married women—carries with it the recognized privilege of demanding removal in all operable tumors of two months' standing after the thirty-fifth year. Though this rule may at times involve the removal of a benign tumor or a chronic inflammatory deposit, there is even then an appreciable advantage to the patient, while deliberating over an always advisable operation has hundreds of times resulted in hopeless progress of the disease.

The pathology of the present establishes beyond doubt that many indolent and seemingly benign tumors, after years of quiescence, suddenly begin to grow, and to manifest the indications and pursue the course of malignancy, whether from the development of latent cells of malignant tendency, or the transformation of previously benign cells into those of malignant character, either carcinomatous or sarcomatous. Hence no operable tumor, even if seemingly innocent, should be allowed to remain after the patient approaches the age at which malignancy threatens. So we regard the diagnosis as almost making itself.

At this stage, within three to five months of the discovery of the tumor, lymphatic involvement, even if it exist in the axilla, is usually not discoverable, and ulceration never is present, nor is the loss of flesh and strength nor cachexic appearance, unless perhaps mental distress has depressed the patient. Here there is absolutely no other response to appeal for treatment but an early and thorough operation. By early is meant *next week;* by thorough is meant removal as suggested by Meyer, Halstead, and Dennis of all *possibly* infected tissue.

It is not here intended to go into any description of the operation.

If, however, when the patient is first seen this hopeful period has passed, and the stage of lymphatic involvement, perhaps ulceration and constitutional depression, has occurred, the question of operation as a conservatism is a judicial rather than a peremptory one. The removal of a depressing and perhaps offensive local lesion, depending on the degree of progress it has made, is often a helpful step, though its results are but temporary relief, and the influence of such failures acts unfavorably on the laity, and prevents such sufferers as may be cured from accepting surgery. The same arguments apply to the removal of recurrences. When after operation local or regional recurrence, more or less isolated and movable, without cachexia appears, prompt and thorough excision is demanded. When, however, the recurrence is accompanied with ulceration and constitutional involvement, the propriety of operative steps is a question of conservatism. Though this is an extensive subject, it is not wise to get deeper into it lest we leave the established facts for speculation, and weaken the value of our conclusions.

Carcinoma of the lip is always of the epithelial variety. The position occasions early discovery, and usually a diagnosis by exclusion can be soon established. Epithelioma of the lip is nearly always seen on the lower labial muco-cutaneous junction, most commonly in middle-aged men, though often seen in women. The use of the short clay pipe and the irritation of broken teeth are factors in causation.

Practically speaking, the diagnosis of the fissure, ulcer, or wart in which this lesion originates can be confounded only with labial chancre. In this latter lesion the ulcer either heals in a few weeks or else becomes a tumor much in excess of the size an epithelioma would reach in the same period. Also the lymphatic involvement of syphilis is early, in the first three weeks, while in carcinoma it is three to six months in succeeding the ulcer. The question of syphilis settled, by observing these points and by the history and age of the patient, as well as the appearance of the ulcer, there is left only the treatment, which should be prompt and free excision. The application of salves and caustics serves only to aggravate the trouble and lose valuable time. The excision promptly done removes the whole trouble while yet local, with a prognosis more favorable than obtains after any other form or location of carcinoma. After glandular infection with constitutional involvement the prognosis and treatment are to be regarded in the same light as that of carcinoma of the breast.

Carcinoma of the penis is also an epithelial variety, beginning as an ulcer, wart, or fissure on the foreskin usually. Rarely is this lesion seen before the forty-fifth year, and commonly after the fiftieth, frequently quite late in life. It grows more rapidly than epithelioma of the lip, from the irritation to which the parts are subjected. Unfortunately its existence is often concealed, from false modesty, or a fear of being misunderstood. The differentiation frcm syphilis is made as in epithelioma of the lip. The destruction of the foreskin and glans progresses sometimes quite rapidly, so that within four to six months they may be almost destroyed by ulceration. Glandular involvement often is delayed beyond what would be expected from the local ravages, and constitutional symptoms are deferred even later yet. The treatment of this condition is early operation, inasmuch as the rapid destruction of tissue involves removal of more of the organ in delay. Complete removal of the organ, even the crura being detached from the pubis, is demanded in neglected cases. The prognosis after early operation is almost if not quite as favorable as in the lip. The same conservatism, however, is due here as in other situations after constitutional symptoms appear.

We conclude, then:

1. That carcinoma, being primarliy a local lesion, demands prompt and wide removal on recognition.

2. The diagnosis of suspected malignancy, should not hesitate to decide for removal of all diseased structures which the body can well spare, even if not confirmed.

3. Conservative operative steps in both primary and recurrent carcinoma are often of highest value.

4. As the mortality after such operative steps as are here recommended is trifling, and death without it certain, no legitimate reason for delay can be offered.—*H. H. Grant, in Louisville Journal of Surgery and Medicine.*

